# Be Strong Enough to Say No: Self-Affirmation Increases Rejection to Unfair Offers

**DOI:** 10.3389/fpsyg.2016.01824

**Published:** 2016-11-22

**Authors:** Ruolei Gu, Jing Yang, Yuanyuan Shi, Yi Luo, Yu L. L. Luo, Huajian Cai

**Affiliations:** ^1^CAS Key Laboratory of Behavioral Science, Institute of Psychology, Chinese Academy of SciencesBeijing, China; ^2^College of Tourism, Huaqiao UniversityQuanzhou, China; ^3^Faculty of Social Science, The Chinese University of Hong KongHong Kong, China; ^4^State Key Laboratory of Cognitive Neuroscience and Learning, Beijing Normal UniversityBeijing, China

**Keywords:** self-affirmation, social decision-making, ultimatum game, fairness, social rejection, event-related potential, P3

## Abstract

We propose that self-affirmation may endow people more psychological resources to buffer against the negative influence of rejecting unfair offers in the classic ultimatum game (UG) and further lead to a stronger tendency to reject those offers. We tested this possibility by conducting an event-related potential (ERP) study about the UG, with the ERP component P3 as an indirect indicator of psychological resources. Participants were randomly assigned to the affirmation or control condition and then completed the UG through electrophysiological recording. As expected, the behavioral data indicated that compared with unaffirmed ones, affirmed participants were more likely to reject unfair UG offers; the electrophysiological data indicated that compared to the unaffirmed, affirmed participants showed a greater P3 in response to the presentation of an offer. These findings suggest that psychological resources may play a role in rejecting others beyond the fairness concern, and additionally shed light on the neural mechanisms underlying self-affirmation.

## Introduction

Social decision-making refers to the act of making decisions in which more than one person is involved. In social decision-making, people concern not only with self-interest but also with the interests of others (Gintis et al., 2003; Ruff and Fehr, 2014). As a result, social decisions in real life may not be consistent with the classic economic principle that people try their best to maximize their personal earnings (Ruff and Fehr, 2014). Indeed, this is the case in the ultimatum game (UG), an important paradigm of social decision-making (Güth et al., 1982, 2001). A typical UG involves two players, including a proposer and a responder. The proposer decides how to split an amount of money between the proposer and responder, and the responder chooses either to accept the offer (the money is divided accordingly) or to reject it (neither player receives anything). Deviating from the principle of reward maximization, an established finding shows that responders are prone to decline offers of less than 30% of the total sum (Thaler, 1988; Güth and Tietz, 1990; Nowak et al., 2000). Given the adage that “something is better than nothing,” why do people tend to reject offers in such a circumstance? This issue has fascinated researchers for decades. The dominant theory proposes that perceived unfairness leads to the rejection of an offer (Fehr and Gachter, 2002). According to this theory, the primary motivation for UG responders to reject an inequitable offer is to punish those who treat them unfairly, thereby enforcing a fair social norm even when the punishment also results in a personal cost (i.e., altruistic punishment; see Gintis et al., 2003; Stallen and Sanfey, 2013). This fairness account is thought to have important evolutionary significance because it promotes cooperation and inhibits selfish behavior (Nowak, 2006).

What concerns us in this research is not the motivation that drives UG rejection. Instead, our focus is the sine qua non for following through with the rejection, which, to our knowledge, is an issue that previous literature has largely neglected. It is well established that making decisions consumes psychological resources (e.g., Pocheptsova et al., 2009; Vohs et al., 2009; Polman and Vohs, 2016). This is particularly true in the rejection of others. In a UG context, rejecting a person’s offer not only means economic loss; more importantly, it invites psychological costs as well. For instance, research shows that rejecting someone causes anxiety and social pain (Zhou et al., 2009), and similarly, ostracizing a person depletes psychological resources (Ciarocco et al., 2001). Indeed, existing theories have documented the need for affiliation as a fundamental motivation for human beings (Maslow, 1968; Baumeister and Leary, 1995). These theories and findings suggest that accepting an unfair offer does not necessarily reflect an economically rational decision to pursue benefit maximization, as suggested by classic economic principles (e.g., Haselhuhn and Mellers, 2005). Rather, such a response might simply be the result of insufficient psychological resources to bear the negative effects of rejecting another person. Inversely put, if a responder possesses enough psychological resources or his/her psychological resources are momentarily strengthened, he/she will be more likely to reject an unfair offer. As far as we know, this hypothesis has not been empirically tested. To fill this gap, we conducted a study to examine the influences of self-affirmation on social decision-making during the UG. We expected that self-affirmation would endow people more psychological resources and consequently increase the tendency of rejecting an unfair offer.

Self-affirmation refers to an act that may demonstrate self-adequacy and further affirm a sense of self-integrity (Steele, 1988; Cohen and Sherman, 2014). A typical operationalization of self-affirmation involves people writing about their core personal values and elaborating on why those values are important to them. By doing so, people can restore extensive psychological resources to cope with stressful problems in a positive and approaching way rather than in a passive and avoiding way (Koole et al., 1999; McQueen and Klein, 2006; Sherman and Hartson, 2010; Cohen and Sherman, 2014). Numerous studies have documented the adaptive function of self-affirmation, such as buffering against various threats, stresses, and life difficulties in diverse domains and cultures (for reviews, Sherman and Cohen, 2006; Cohen and Sherman, 2014). In particular, self-affirmation counteracts self-depletion (Schmeichel and Vohs, 2009). Our previous research showed that self-affirmation functions similarly in China as it does in the West (Cai et al., 2013). Unlike in the West, the operative component in China is the affirmation of a familial self (writing about core values shared by oneself and his/her family) rather than the individual self (writing about personal core values; Cai et al., 2013).

In this study, we asked Chinese participants to affirm their familial self or not and then to complete a trial-by-trial UG task. China is a representative culture of collectivism, where interpersonal harmony is highly valued (Markus and Kitayama, 1991; Zhang et al., 2005). As a result, rejecting a person would be particularly difficult and entails substantial psychological resources (Pöhlmann et al., 2007), which makes China an ideal place to test our hypotheses. We infer that self-affirmation would momentarily equip people with more psychological resources and ultimately make Chinese participants more likely to reject an unfair offer. To test this behavioral impact of self-affirmation, we used the rejection rate of UG offers as an index. We predicted that the rejection rate of unfair offers in the self-affirmation condition would be higher than the control condition; for fair offers, however, we expected no significant difference in rejection rate because there is no motivation for people to reject a fair offer.

To support our psychological resource account in explaining the rejection of unfair offers, we need to demonstrate that self-affirmation would endow people more psychological resources momentarily. Since there is no direct behavioral measurement of psychological resources (Sherman, 2013), we relied on the event-related potentials (ERP) instead. In UG studies, two ERP components are frequently used: the feedback-related negativity (FRN) and P3 (Boksem and De Cremer, 2010; Luo et al., 2014). The FRN is a fronto-central negativity that peaks at approximately 200–300 ms post-onset of outcome feedback (Gehring and Willoughby, 2002). In non-social decision-making tasks, the FRN automatically encodes the economic value of an event, such that monetary losses elicit a larger FRN than gains (San Martín, 2012, for a review). Similarly, in social decision-making tasks such as the UG, an unfair offer elicits a larger FRN than a fair offer (Boksem and De Cremer, 2010; Van der Veen and Sahibdin, 2011; Alexopoulos et al., 2012). In this sense, the FRN is considered to be an automatic monitor of fairness (Luo et al., 2014). Following the FRN, the P3 is a centro-parietal positivity appearing in the 300–600 ms time window, which also plays an important role in outcome evaluation (San Martín, 2012). Specifically, the P3 amplitude represents the extent to which cognitive resources are involved in outcome evaluation; a larger P3 indicates more cognitive resources such as attentional and execute control resources are allocated to the current scenario (Kok, 1997; Utku et al., 2002; Polich, 2007). Indeed, numerous studies have shown that in performing a task, the more cognitive resources are implicated, the larger P3 component will be observed (Olofsson et al., 2008; Polezzi et al., 2010). In this study, we suggest that the P3 could be regarded as an indirect indicator of psychological resources. When psychological resources are depleted, people would be more likely to make decisions based on gut feelings (Levav et al., 2010), thereby minimizing the cognitive resources needed in outcome evaluation; in this case, a smaller P3 would be expected. Inversely put, when psychological resources are momentarily increased, people would invest more cognitive resources and make more thoughtful decisions (Tice et al., 2007; Wang et al., 2010; DeWall et al., 2011); in this case, an enlarged P3 would be expected. We predicted that self-affirmation would lead to an enlarged P3 during the evaluation of an offer, regardless of its fairness level. We also examined the FRN amplitude. Since self-affirmation affects UG behavior by modulating the amount of psychological resources rather than fairness perception, we predicted that self-affirmation would not produce any discernible difference in the FRN.

## Materials and Methods

### Participants

We recruited 40 Chinese college students online to participate in the experiment. These students came from Beijing-based universities located near the Institute of Psychology, Chinese Academy of Sciences, such as Beijing Forestry University and China Agricultural University. One student declined our invitation. Another student bowed out of the experiment because of health issues. As a result, the final sample consisted of 38 participants in total, who were randomly assigned to the familial self-affirmation condition and the control condition, such that both groups were composed of 19 participants (affirmation condition: 13 females; control condition: 8 females). An independent-sample *t*-test revealed that the participants in the two conditions did not differ significantly in age (affirmation condition: 22.89 years, control condition: 24.37 years; *t*(36) = 1.76, *p* = 0.087).

All participants reported that they were free of regular use of any substance that might influence the central nervous system; none had a history of neurological disease. All had normal vision (with correction) and were right-handed. Also, all participants submitted their written informed consent prior to the experiment. The Institutional Review Board (IRB) in the Institute of Psychology, Chinese Academy of Sciences approved the experimental protocol.

### Procedure

The self-affirmation procedure was derived from Cai et al. (2013). In the self-affirmation condition, participants chose one value that they and their family cherished most from four domains (financial wealth, social network, art/creativity, and knowledge). Then they were asked to record their explanation about why their chosen value was important to them and their family (no less than 150 words). Finally, they were required to describe an experience in which they realized how important this value was to them and their family (no less than 150 words). In the control condition, participants chose a value that was least important to them, recorded why this value might be important to ordinary students, and described an experience in which they realized that this value was important to ordinary students^1^.

Immediately afterward, participants in both conditions were informed of the UG rules, of which the design replicated that of Luo et al. (2014). Participants were told that the affirmation manipulation and the UG task belonged to different research projects for different purposes. To reinforce the social nature of the task, participants were told that they would play the UG together with three other anonymous college students. In actuality, no one else was playing the game, and participants received no further information about the identities of the supposed players. Participants were also told that they would be assigned to different roles (proposer or responder) to be determined by drawing lots prior to the task. In reality, all participants received instruction to play responder.

**FIGURE 1:**
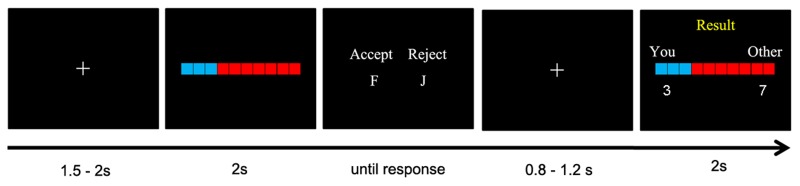
**Schematic depiction of a single trial setting.** In this exemplar trial, the responder accepts the offer proposed by another player, thus the money is split between the two as proposed. Note that in the original task, all the verbal information presented was written in Chinese.

In the formal task, participant sat comfortably approximately 100 cm in front of a computer screen. Stimulus display and behavioral data acquisition were conducted using E-Prime 2.0 (Psychology Software Tools, Inc.). Each trial began with the presentation of a central fixation cross for 1.5–2 s (randomized across trials). Thereafter, either a person posing as a player or the computer proposed an offer (8.5° × 1.5°) for an interim of 2 s to split 10 Chinese Yuan (~1.5 US dollars) with participants. As responders, participants decided whether or not to accept the offer by pressing the F or J buttons on the keyboard with their left or right index fingers (the button assignments were counterbalanced across participants). After participants made their decisions, they waited for 0.8–1.2 s to receive feedback, which informed them of the results of the current trial (**Figure 1**).

Before the task, participants were informed that their compensation for the experiment would be 20 Chinese Yuan plus the cumulative outcome of the UG. The higher the score a participant earned, the higher the payment they would receive at the end of the experiment. Participants were also told that they would finish the task with three other anonymous college students; that the offer in each trial was selected randomly from the other three players; that they would be unable to identify which proposer suggested the offer in a given trial; and that the proposers would not know whether the participants accepted or rejected their offers (Boksem and De Cremer, 2010). Unbeknownst to the participants, all offers were actually assigned by the computer in predetermined pseudorandom sequences. The task contained three blocks in total, each of which consisted of 50 trials, with each block separated by a short interval. Each block included 20 equitable trials (10 offers of 50:50 and 10 of 40:60), 20 inequitable trials (10 offers of 10:90 and 10 of 20:80), and 10 moderate inequitable offers (30:70). After participants finished the task, they were debriefed and remunerated with 100 Chinese Yuan regardless of their performance. According to their self-report, all the participants believed that they were interacting with human proposers during the task.

### Electrophysiological Recording and Preprocessing

An electroencephalogram (EEG) was recorded from 64 scalp sites using tin electrodes mounted in an elastic cap (NeuroScan Inc.) with an online reference to the left mastoid and off-line algebraic re-reference to the average of the left and right mastoids. In addition, an electrooculogram (EOG) was recorded for the purpose of artifact correction. Horizontal EOG was recorded from electrodes placed at the outer canthi of both eyes and Vertical EOG from electrodes placed above and below the left eye. All inter-electrode impedance was maintained at <5 kΩ. EEG and EOG signals were amplified with a 0.05–100 Hz online band-pass filter and continuously sampled at 500 Hz/channel.

During the off-line analysis, ocular artifacts were removed from the EEG signal using a regression procedure implemented with Neuroscan software (Semlitsch et al., 1986). After 0.05–30 Hz band-pass digital filtering through a zero phase shift, the EEG data were segmented into epochs time-locked to the onset of the offer presentation. Separate EEG epochs of 1000 ms were baseline-corrected by subtracting from each sample the average activity of that channel during the -200–0 ms baseline period. Any trial in which EEG voltages exceeded a threshold of ± 100 μV during the recording epoch was excluded from further analysis. After the data preprocessing described above, the trials survived were determined as artifact-free (fair condition: 94.4 ± 2.5% of the 60 trials; unfair condition: 94.1 ± 2.5% of the 60 trials).

### Data Analysis

The rejection rates for fair (i.e., 40:60 and 50:50) and unfair (i.e., 10:90 and 20:80) offers were calculated, respectively (Hewig et al., 2011; Harle and Sanfey, 2012). Consistent with our previous studies (T. Wu et al., 2013; Luo et al., 2014), moderate unfair offers (30:70) were excluded from data analysis, because UG players often disagree on whether such offers should be regarded as fair or not (Halko et al., 2009). Therefore, it is difficult to categorize this kind of offer. In addition, excluding moderate unfair offer also makes the data analysis more parsimonious.

Previous literature suggests two ways to calculate the FRN amplitude, that is, either using grand-averaged ERPs or creating a difference wave between “error” and “correct” trials (Holroyd et al., 2008; Wu and Zhou, 2009). We measured the grand-averaged waveforms in this study because the difference wave approach is unsuited for exploring whether self-priming affected neural response in the unfair condition, the fair condition, or both (see also Luo et al., 2014). The amplitudes of the FRN and P3 were calculated as the mean values within the 250–350 and 400–600 ms time windows following the presentation of the UG offer, respectively. The time windows were selected through visual inspection of grand-averaged waveforms. The electrodes in which the ERP components reached their maximum were chosen for further analysis (see the “*ERP Results”* subsection).

Rejection rates, FRN amplitudes, and P3 amplitudes were analyzed using two-way Fairness (fair vs. unfair) × Self-affirmation (self-affirmation condition vs. control condition) ANOVA tests, with Self-affirmation as the between-subject factor. For all the analyses, the significance level was set at 0.05. Significant effects were analyzed using simple-effect models (LSD, two-tailed). Partial eta-squared ($\eta_{p}^{2}$) values were reported to examine the size of effects in ANOVA models.

## Results

### Behavioral Results

**FIGURE 2:**
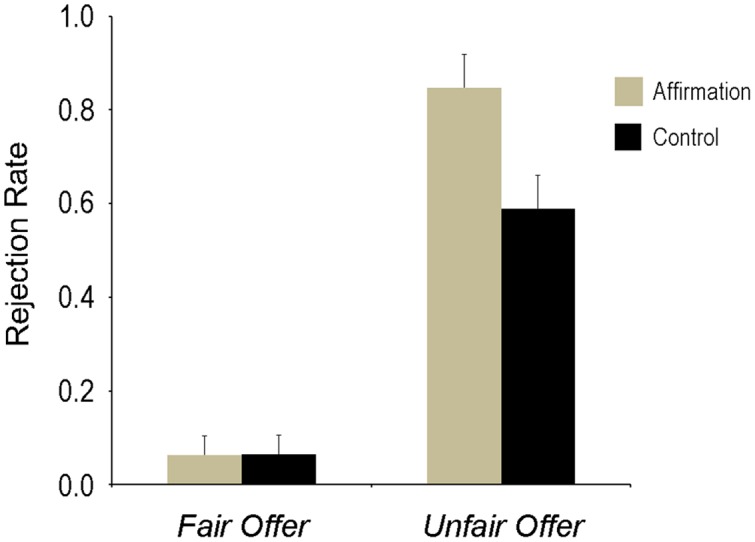
**Rejection rate of offers.** Error bars indicate 1 *SE*.

The main effect of the Fairness was significant, *F*(1,36) = 161.33, *p* < 0.001, $\eta_{p}^{2}$ = 0.82; the rejection rate was higher for unfair offers than fair offers (71.8 ± 5.1% vs. 6.4 ± 2.9%). The main effect of Self-affirmation was marginally significant, *F*(1,36) = 3.97, *p* = 0.054, $\eta_{p}^{2}$ = 0.10; the rejection rate showed a tendency to be higher in the self-affirmation condition than in the control condition (45.5 ± 4.6% vs. 32.7 ± 4.6%). This effect was qualified by Fairness as indicated by a significant Fairness × Self-affirmation interaction (**Figure 2**), *F*(1,36) = 6.30, *p* = 0.017, $\eta_{p}^{2}$ = 0.15; participants in the self-affirmation condition were more likely to reject unfair offers than in the control condition (84.7% vs. 58.9%; *p* = 0.015), while no significant difference was found for fair offers (6.3% vs. 6.4%; *p* = 0.988). Our hypothesis on behavioral data was confirmed.

### ERP Results

#### The FRN

**FIGURE 3:**
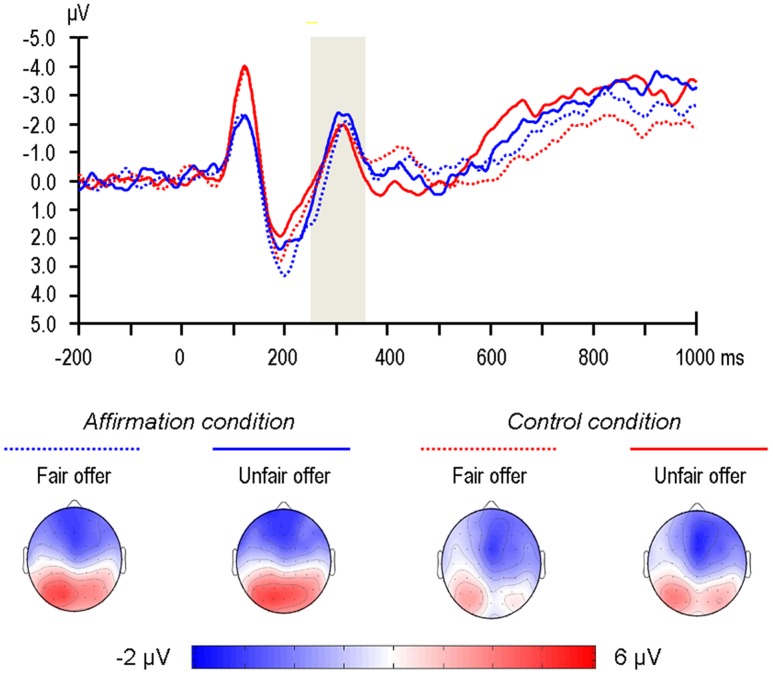
**Grand-average event-related potentials (ERPs) evoked by offer presentation at the Fz recording site, where the feedback-related negativity (FRN) was measured.** The time point 0 indicates the onset of offer presentation. The shaded gray areas indicate the 250–350 ms time window for the calculation of the mean value of the FRN. The scalp topographies of each condition are presented beneath.

According to visual detection on the scalp topographies, the FRN was determined to be maximal in the fronto-central area (**Figure 3**). Accordingly, the arithmetic means of three electrodes in this area (Fz, FCz, Cz) were calculated for further analysis. Neither the main effect of Fairness, *F*(1,36) = 0.05, *p* = 0.82, $\eta_{p}^{2}$ < 0.01, nor the main effect of Self-affirmation, *F*(1,36) = 0.24, *p* = 0.63, $\eta_{p}^{2}$ = 0.01, nor the Fairness × Self-affirmation interaction, *F*(1,36) = 0.80, *p* = 0.38, $\eta_{p}^{2}$ = 0.02, was significant (**Figure 3**).

#### The P3

**FIGURE 4:**
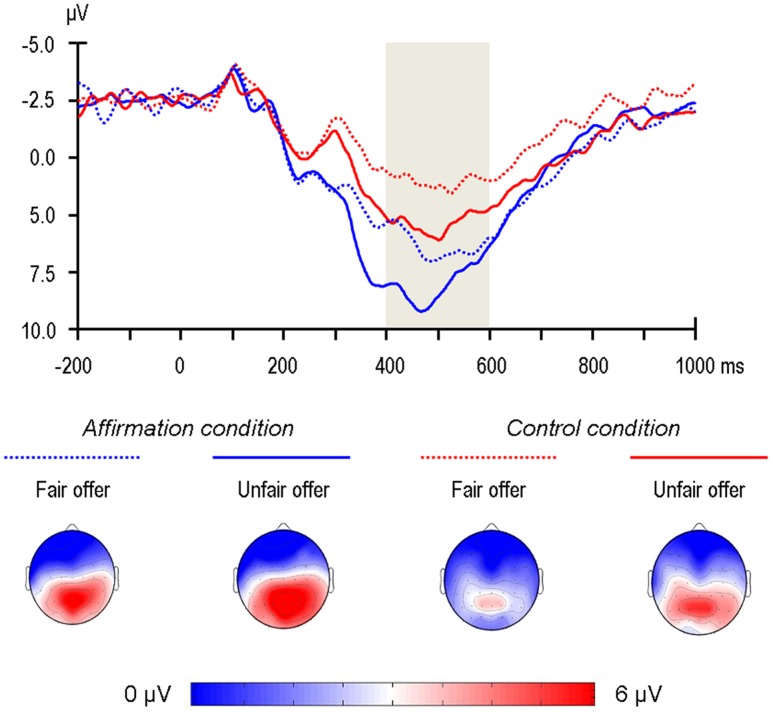
**Grand-average ERPs evoked by offer presentation at the Pz recording site, where the P3 component was measured.** The time point 0 indicates the onset of offer presentation. The shaded gray areas indicate the 400–600 ms time window for the calculation of the mean value of the P3. The scalp topographies of each condition are presented beneath.

According to visual detection on the scalp topographies, the P3 was determined to be maximal in the centro-parietal area (**Figure 4**). Accordingly, the arithmetic means of three electrodes in this area (Cz, CPz, Pz) were calculated for further analysis. The main effect of Fairness was significant, *F*(1,36) = 10.56, *p* = 0.003, $\eta_{p}^{2}$ = 0.23; unfair offers elicited a larger P3 than fair offers (5.02 μV vs. 3.70 μV). Most interesting, the main effect of Self-affirmation was significant, *F*(1,36) = 4.79, *p* = 0.035, $\eta_{p}^{2}$ = 0.12; the P3 was larger in the self-affirmation condition than in the control condition (5.41 μV vs. 3.32 μV; see **Figure 4**), which held true under both the fair and unfair conditions as indicated by the non-significant Fairness × Self-affirmation interaction, *F*(1,36) = 0.01, *p* = 0.91, $\eta_{p}^{2}$ < 0.01. Our hypothesis on the P3 was confirmed.

## Discussion

We conducted an ERP study to examine the influence of self-affirmation on social decision-making in the UG context. Insofar as we know, this is the first study to examine the impacts of self-affirmation on social decision-making. Consistent with previous findings, participants were more likely to reject unfair offers than fair offers (Thaler, 1988; Güth and Tietz, 1990; Nowak et al., 2000). Most importantly, self-affirmation influences both behavioral and brain responses. At the behavioral level, we found that when the offer was fair, the rejection rate in the self-affirmation condition did not differ significantly from the control condition; however, when the offer was unfair, the rejection rate in the self-affirmation condition was higher than that in the control condition. At the neural level, we found that the P3 amplitude was significantly larger in the self-affirmation condition than in the control condition regardless of the fairness of the offer.

Our findings shed light on the understanding of a prevalent phenomenon in social decision-making: people tend to reject an offer in UG, even at the price of an economic loss. The dominant fairness account suggests that when an offer is perceived to be unfair, people tend to reject it at the expense of certain personal benefits in order to maintain a fair world. Given that rejecting an offer may incur not only economic but also psychological costs (Baumeister and Leary, 1995; Zhang et al., 2009), we propose that whether a person has enough psychological resources to cope with the negative influences of rejecting others also plays a role in his/her social decision-making. If a person’s psychological resources and concomitant tolerance of the consequences of rejection are temporarily strengthened, he/she will be more likely to reject an unfair offer. We capitalize on self-affirmation, a self-regulation strategy that may help people to draw psychological resources to cope with stressful situations. Results based on the neural index P3 indicate that self-affirmation boosts participants’ psychological resources momentarily. Additionally, results based on behavioral responses indicate that affirmed participants are more likely to reject unfair offers than control participants. To sum up, the findings indicate that self-affirmation enables more psychological resources available and increase rejection of unfair offers accordingly. However, it is worth noting that we have used a neural measure for psychological resource because behavioral measure is not available so far.

We have capitalized on self-affirmation as a way to boost psychological resources so that people are more likely to reject an unfair offer. The current results support this proposal and are also consistent with a past finding that self-affirmation offsets depletion of psychological resources (Schmeichel and Vohs, 2009). Given that many mediators have been proposed to explain self-affirmation effects and many consequences of self-affirmation have been identified (Sherman and Cohen, 2006), people may suggest that the influence of self-affirmation on UG performance manifested via other mechanisms rather than boosting psychological resources. For example, self-affirmation may function by boosting state self-esteem, promoting positive affects, enhancing a transcendental perspective, or reducing cognitive dissonance (Sherman and Cohen, 2006; Cohen and Sherman, 2014). However, we would like to point out that these theories are not conflicting with the psychological resource account because essentially they are all manifestations or consequences of increased psychological resources (Tesser, 2000; Sherman and Cohen, 2006; Cohen and Sherman, 2014). Hence it is not surprising that they may also explain our findings to some extent. For instance, the broadened view may make people less concern about the negative effect of rejecting the proposer. However, it would be difficult for this theory to explain why the P3 component was enlarged rather than decrease. In contrast, the psychological resource account provides a more direct and parsimonious interpretation for the ERP findings.

An alternative interpretation is that self-affirmation affected subjective fairness perception of UG proposals, which further resulted in a higher rejection rate of unfair offers among affirmed people. In this case, it would be difficult to explain why the FRN amplitude, an index of fairness judgment (Boksem and De Cremer, 2010; Van der Veen and Sahibdin, 2011; Alexopoulos et al., 2012; Luo et al., 2014), was insensitive to self-affirmation. Another alternative interpretation is that self-affirmation made people blind to the proposer’s feelings. Recent fMRI studies, however, have found that self-affirmation produces heightened activity in the ventromedial prefrontal cortex (vmPFC; Falk et al., 2015; Cascio et al., 2016), which is a brain area crucial for empathy (Botvinick et al., 2005). These findings suggest that affirmed participants might know better about how the proposers felt than unaffirmed participants. Even so, they still chose to reject the offers. Future studies may include a measure to assess how people perceived proposer’s feelings and test this possibility directly.

Our finding that affirmed people are more likely to reject an unfair offer may help understand some past findings in a novel perspective. For instance, Campanha et al. (2011) identified an attenuation of altruistic punishment (i.e., punishing unfair offers) among participants when playing UG with a friend over playing with a stranger. The classic fairness account suggests that friendship may have lessened the perceived unfairness of the offer and resulted in fewer rejections of an unfair offer (Campanha et al., 2011). We suggest another possibility: participants may choose not to reject an unfair offer due to concerns about the damage to their friendship with the proposer (see also Campanha et al., 2011). Inversely put, if participants had enough psychological resources such that they did not care about the negative consequences of rejection, they may not have refrained from rejecting the offer. Future studies could directly examine the possibility that self-affirmation enables people to say “no” to an unfair proposal from a friend.

Our study also contributes to the literature on self-affirmation in several ways. First, previous research has demonstrated the influences of self-affirmation on various psychological processes (for reviews, Sherman and Cohen, 2006; Cohen and Sherman, 2014). For the first time, we find out that self-affirmation may also impact social decision-making by increasing the possibility of rejecting an unfair offer, thus extending the understanding of the functions of self-affirmation. Second, our study also sheds light on the neural mechanism underlying self-affirmation. Burgeoning research has started to investigate the influence of self-affirmation on brain responses. A first ERP study showed that self-affirmation enlarges the error-related negativity (ERN) component, the amplitude of which further predicts performance errors, suggesting that self-affirmation increases the openness to mistakes via enhanced error-monitoring (Legault et al., 2012). A recent fMRI study indicated that self-affirmation increases the neural activity of vmPFC in response to health risk information, which in turn predicts declines in sedentary behavior (Falk et al., 2015). These findings suggest that self-affirmation reduces defensiveness by viewing otherwise threatening information as self-relevant. Latest brain-imaging studies have also demonstrated that self-affirmation heightens the activity of the ventral striatum, suggesting that self-affirmation may be adaptive due to its rewarding function (Cascio et al., 2016; Dutcher et al., 2016). Adding to this wealth of findings, the current study indicates that self-affirmation increases psychological resources as reflected by the enlarged P3 during social evaluation. Taken together, these studies illustrate the neural underpinnings of self-affirmation from multiple perspectives (Steele, 1988; Sherman and Cohen, 2006; Cohen and Sherman, 2014).

The FRN represents an early encoding of the monetary outcome of the offer, which is supposed to be automatic and independent of psychological resources (D. Zhang et al., 2014; Yang et al., 2015). Consistent with our hypothesis, the FRN was not sensitive to self-affirmation. Unexpectedly, the FRN did not vary with the fairness level of the UG offer. Given that past research has consistently shown that unfair offers elicit a larger FRN than fair offers (Boksem and De Cremer, 2010; Luo et al., 2014), it is inappropriate for us to make any particular speculations before ensuring that this unusual finding is replicable. Although the insensitivity of FRN to self-affirmation may suggest that self-affirmation does not affect the rejection to unfair offers by influencing fairness perception, its insensitivity to high versus low fairness dampens this speculation. Thus, future replications are needed.

In summary, this study suggests that self-affirmation augments psychological resources in social decision-making and further increase the rejection of an unfair offer. Our findings, however, are preliminary due to several reasons. First, people may expect that the change in the P3 could mediate the influence of self-affirmation on the behavioral rejection rate of unfair offers. Actually, we have examined this possibility but failed to find any significant mediating effect (not included in the manuscript). Second, we have hypothesized that the concerns about interpersonal relationship play an important role in social decision-making. However, we did not include any measure (e.g., sensitivity to reject others) to verify this possibility. Future study may include relevant measures and test this hypothesis directly. Third, although the sample size in our study is comparable to similar ERP studies (e.g., Legault et al., 2012; Luo et al., 2014), it is relatively small compared to typical behavioral studies on self-affirmation (e.g., Schmeichel and Martens, 2005; Schmeichel and Vohs, 2009). Future study may use larger sample. Fourth, we have conducted our study in China, a representative collectivistic culture, where interpersonal harmony is highly valued and the interpersonal harmfulness in rejecting an offer poses a real concern (Markus and Kitayama, 1991). We do not know if our findings would also hold true beyond China. Finally, we have only examined one paradigm of social decision-making and do not know if the findings would also apply to other paradigms, such as the prisoner’s dilemma. Future replications in different cultures and with different paradigms are needed.

## Authors Contributions

RG, JY, and HC conceived and designed the experiments. JY and YS performed the experiments. JY, YiL, and HC contributed materials and analysis tools. RG and JY analyzed the data. RG, JY, YuL, and HC wrote the manuscript. The authors thank Ziyan Yang for help with manuscript revision.

## Conflict of Interest Statement

The authors declare that the research was conducted in the absence of any commercial or financial relationships that could be construed as a potential conflict of interest.

## Footnotes

1 In the affirmation condition, financial wealth, social network, and knowledge were chosen by six participants as the most important value, respectively; one participants chose art/creativity. In the control condition, 16 participants chose art/creativity as the least important value, two chose financial wealth, and one chose social network.
